# Placental Antioxidant Defenses and Autophagy-Related Genes in Maternal Obesity and Gestational Diabetes Mellitus

**DOI:** 10.3390/nu13041303

**Published:** 2021-04-15

**Authors:** Cecilia Diceglie, Gaia Maria Anelli, Cristina Martelli, Anais Serati, Alessia Lo Dico, Fabrizia Lisso, Francesca Parisi, Chiara Novielli, Renata Paleari, Irene Cetin, Luisa Ottobrini, Chiara Mandò

**Affiliations:** 1 Department of Pathophysiology and Transplantation, Università degli Studi di Milano, 20054 Segrate, Italy; cecilia.diceglie@unimi.it (C.D.); cristina.martelli@unimi.it (C.M.); anais.serati@unimi.it (A.S.); alessia.lodico@unimi.it (A.L.D.); renata.paleari@unimi.it (R.P.); 2 Department of Biomedical and Clinical Sciences “Luigi Sacco”, Università degli Studi di Milano, 20157 Milano, Italy; gaia.anelli@unimi.it (G.M.A.); fabrizia.lisso@unimi.it (F.L.); chiara.novielli@unimi.it (C.N.); irene.cetin@unimi.it (I.C.); 3 Department of Woman, Mother and Child, Luigi Sacco and Vittore Buzzi Children Hospital, ASST Fatebenefratelli-Sacco, Università degli Studi di Milano, 20154 Milano, Italy; parisi.francesca@asst-fbf-sacco.it; 4 Institute of Molecular Bioimaging and Physiology, National Research Council (IBFM-CNR), 20054 Segrate, Italy

**Keywords:** autophagy, pregnancy, maternal obesity, GDM, placenta, macroautophagy, chaperone-mediated autophagy (CMA), antioxidant defenses, sexual dimorphism

## Abstract

Maternal obesity and gestational diabetes mellitus (GDM) are increasing worldwide, representing risk factors for both mother and child short/long-term outcomes. Oxidative stress, lipotoxicity and altered autophagy have already been reported in obesity, but few studies have focused on obese pregnant women with GDM. Antioxidant and macro/chaperone-mediated autophagy (CMA)-related gene expressions were evaluated herein in obese and GDM placentas. A total of 47 women with singleton pregnancies delivered by elective cesarean section were enrolled: 16 normal weight (NW), 18 obese with no comorbidities (OB GDM(–)), 13 obese with GDM (OB GDM(+)). Placental gene expression was assessed by real-time PCR. Antioxidant gene expression (*CAT*, *GPX1*, *GSS*) decreased, the pro-autophagic *ULK1* gene increased and the chaperone-mediated autophagy regulator *PHLPP1* decreased in OB GDM(–) vs. NW. On the other hand, *PHLPP1* expression increased in OB GDM(+) vs. OB GDM(–). When analyzing results in relation to fetal sex, we found sexual dimorphism for both antioxidant and CMA-related gene expressions. These preliminary results can pave the way for further analyses aimed at elucidating the placental autophagy role in metabolic pregnancy disorders and its potential targetability for the treatment of diabetes outcomes.

## 1. Introduction

Maternal obesity (MO) is expanding exponentially worldwide to almost epidemic proportions, representing a significant risk factor for negative pregnancy outcomes, with short- and long-term consequences for both the child and the mother [1,2]. Gestational diabetes mellitus (GDM) is the most frequent complication of pregnancy, presenting in 2–14% of cases [3]. Up to 50% of obese women (OB) develop GDM during pregnancy, with an increased risk for the mother to later develop type 2 diabetes. Moreover, the adverse intrauterine environment of MO and GDM alters the programming of fetal metabolic functions [4], yielding possible intergenerational effects [5], thus perpetuating a vicious cycle that has become a major public health concern [1].

MO and GDM have been associated with a specific placental phenotype characterized by low-grade inflammation, increased oxidative stress (OxS) and release of reactive oxygen species (ROS) from placental mitochondria (mt) [6]. We recently reported increased mt DNA levels in placentas of OB, further characterized by marked dysfunctional morphology in GDM [7].

Autophagy is a dynamic mechanism employed by cells to get rid of altered proteins or macromolecules and defective organelles. It allows the maintenance of cell homeostasis under environmental stress, cooperating with the main enzymes of the antioxidant system, physiologically reducing cellular ROS levels [8].

Autophagic mechanisms are known to be induced in response to nutrient limitation or to OxS and inhibited by an excess of amino acids and growth factors or insulin altering the intracellular milieu [9]. These cytotoxic insults interfere with the autophagic regulation depending on the tissue type [10].

Autophagy includes different aspecific and specific processes. Macroautophagy is involved in the non-specific degradation of different cellular components. On the other hand, chaperone-mediated autophagy (CMA) is responsible for the degradation of specific cytosolic proteins inside lysosomes, and it has been reported to be necessary for cell homeostasis [11,12]. Autophagic processes can be triggered by OxS [13], also in its genotoxic form [14], as well as metabolic stress [15], intracellular glucose excess [16] and hypoxia [17,18].

Autophagy in pregnancy is becoming a new challenge of research [17,18,19]. Inadequate regulation of the ROS–autophagy axis in early pregnancy leads to impaired autophagy activity and contributes to the development of preeclampsia and intrauterine growth restriction, two pregnancy pathologies characterized by placental insufficiency [9]. However, very few studies have focused on placental autophagy in relation to obesity with or without GDM [19,20,21]. Moreover, as far as we know, no evidence of chaperone-mediated autophagy is described for human placentas.

In this paper, we aim to analyze placental molecular events related to autophagy in the context of metabolic disorders of pregnancy, to elucidate any possible relation between clinical features and this complex pathway, with the purpose of understanding new pathogenic deregulations and identifying new potential therapeutic targets.

## 2. Materials and Methods

### 2.1. Population

Pregnant women were enrolled in the Obstetric Units of the L. Sacco Hospital and the V. Buzzi Children Hospital (ASST Fatebenefratelli-Sacco) in Milan.

The study was conducted in accordance with the Declaration of Helsinki and in compliance with all current Good Clinical Practice guidelines, local laws, regulations and organizations. The protocol was approved by the hospital ethical committee (Prot. N. 17739/2018). All participants gave their informed consent to collect personal data and biological samples.

Forty-seven Caucasian pregnant women with single-term pregnancies were prospectively recruited at elective cesarean section. The study population was divided according to pre-pregnancy body mass index (BMI, kg/m^2^), following the 2009 Institute of Medicine (IOM) guidelines [22]:

1. Normal Weight (NW) women: 18 ≤ BMI < 25, *n* = 16;
2. Obese (OB) women: BMI ≥ 30, *n* = 31.

According to the protocols in use, counselling was provided for dietary and lifestyle intervention to reach the appropriate gestational weight gain (GWG, kg) according to pregestational BMI and as recommended by the IOM [22]:

1. NW women: 11.5 ≤ GWG ≤ 16;
2. OB women: 5 ≤ GWG ≤ 9.

Gestational diabetes mellitus (GDM) was diagnosed in a subgroup of OB women (OB GDM(+), *n* = 13) according to the International Federation of Gynecology and Obstetrics (FIGO) guidelines, by the Oral Glucose Tolerance Test (OGTT-75 g) performed at 24–28 weeks of gestation [23,24]. The OGTT procedure requires a fasting blood glucose test (OGTT I) and then glycemia checks after 60 min (OGTT II) and after 120 min (OGTT III) from the administration of 75 g of anhydrous glucose dissolved in 300 mL of water. Blood glucose quantification at each time point was performed after venous blood withdrawal and standardized clinical biochemistry laboratory dosage by enzymatic spectrophotometric analysis (the hexokinase/glucose-6-phosphate dehydrogenase (g6pd) method). GDM was diagnosed for one or more glycemic curve values higher than 92,180,153 mg/dL [23].

Mothers presenting comorbidities different from GDM (i.e., hypertension, autoimmune diseases) or other pregnancy complications (e.g., preeclampsia, infections, congenital/genetic abnormalities) were excluded.

### 2.2. Placental Tissue Sample Collection

Human placentas were collected immediately after elective cesarean section, cleaned of excess blood and then weighed after discarding the membranes and cord from the disc. Biometric measurements were performed as previously described [25,26]. Briefly, placental area was estimated by calculating the area of an ellipse from the diameters (D × d × π/4). Assuming constant density, placental thickness was obtained as weight divided by area. Placental efficiency was calculated as the ratio between fetal weight and placental weight.

Chorionic villi biopsies of 1 cm^3^ were collected in different sites of the placental disc (central, median and peripheral) from the maternal side, after discarding the maternal decidua. Placental tissue samples were carefully washed in phosphate-buffered saline to eliminate excessive blood and conserved in RNAlater at −80 °C.

### 2.3. Gene Expression Analysis

Placental tissue samples were mechanically shredded in a Potter homogenizer with *TRI Reagent* and total RNA was extracted from the tissue homogenate using the column-based *RiboPure Kit* (ThermoFisher Scientific Baltics UAB, Vilnius, Lituania) following the manufacturer’s instructions. RNA concentration was determined spectrophotometrically by *NanoDrop ND-1000* (NanoDrop Technologies, Wilmington, DE, USA), and then 2 μg of RNA were retrotranscribed using the *GoScript Reverse Transcription Mix*, *Random Primers* (Promega, Madison, WI, USA), following the manufacturer’s instructions. For the real-time assay, 4 ng of cDNA were used for each sample amplification.

The expression of antioxidant defenses (CATalase-*CAT*), Glutathione SynthetaSe-*GSS*, Glutathione ReductaSe-*GSR*, Glutathione PeroXidase 1-*GPX1*), macroautophagy (Unc-51-Like Kinase 1-*ULK1*, BECliN-*BECN1*), chaperone-mediated autophagy (Heat Shock Cognate 70 protein-*HSC70*), Lysosomal Associated Membrane Protein 2A (*LAMP-2A*) and Pleckstrin Homology domain and Leucine-rich repeat Protein Phosphatase 1 (*PHLPP1*) and autophagy-related (Nuclear factor erythroid 2-Related Factor 2-*NRF2*), Hypoxia-Inducible Factor 1 (*HIF-1α*), Vascular Endothelial Growth Factor (*VEGF*) genes was quantified by real-time PCR (*7500 Fast Real-Time PCR System*, *Applied Biosystem*) with SYBR Green chemistry (Promega, Madison, WI, USA), in triplicate. Primers and amplicon details are shown in Table 1.

Gene expressions were obtained by a normalization strategy that allows using two different housekeeping genes simultaneously and takes into account the amplification efficiency of each assay in the specific investigated tissue. Specifically, E^−ΔCq^/NF was determined, where E is the amplification efficiency of each assay (calculated by a calibration curve) and NF is the normalization factor. This normalization factor was calculated from the geometric mean of two selected housekeeping genes [27], β-actin (*Beta-actin*) and YWHAZ (*Tyrosine 3-Monooxygenase/Tryptophan 5-Monooxygenase Activation Protein Zeta*).

In more detail, for each sample and assay, the relative quantity Q was calculated:

Q = E^−ΔCq^, where E is the amplification efficiency of each assay and

ΔCq = sample Cq—reference Cq (the reference is a chosen subject, i.e., the one showing the lowest Cq value).

The normalization factor (NF) was then calculated from the geometric mean (GM) of the relative quantities (Q) of the two endogenous control genes:

$$\mathrm{NF}=\frac{\mathrm{GM}\;\mathrm{of}\;b-actin\;\mathrm{and}\;YWHAZ\;\mathrm{relative}\;\mathrm{quantities}\;\left(Q\right)\;\mathrm{for}\;\mathrm{each}\;\mathrm{sample}}{\mathrm{GM}\;\mathrm{of}\;\mathrm{all}\;\mathrm{the}\;\mathrm{geometric}\;\mathrm{means}}$$

Lastly, for each sample, the expression of target genes was calculated by $\frac{Q}{\mathrm{NF}}$.

Only Cq values with standard deviation ≤0.25 across triplicates were included in the statistical analysis.

### 2.4. Statistical Analysis

Maternal characteristics, placental and fetal data and gene expression values were compared among groups by One-way ANOVA or the Kruskal–Wallis test according to data distribution (assessed by the Kolmogorov–Smirnov test). In post hoc analyses, Tukey’s HSD test and the Mann–Whitney U test with Bonferroni correction (thus considering statistical significance when *p* ≤ 0.017, as three groups were analyzed) were used.

Frequencies of placental efficiency, using its median value (6.9) as cut-off, and of fetal sex were evaluated among population groups by performing the chi-square test.

A Two-way ANOVA was conducted to explore the impact of maternal characteristics (pregestational BMI and hyperglycemia) and fetal sex on fetal and placental parameters and gene expression.

Comparisons between the two subgroups of placentas with different efficiency were performed using the independent sample *t*-test or the Mann–Whitney U test according to data distribution.

Correlations between variables were assessed using the Spearman rank order correlation.

Differences and correlations were considered significant when *p* < 0.05.

Analyses were performed using the statistical package SPSS, v.26 (IBM; Armonk, NY, USA).

## 3. Results

### 3.1. Clinical Data

Table 2 summarizes maternal, fetal and placental data of the three study groups.

OB GDM(−) women were significantly younger than the other mothers.

Following defined inclusion criteria, both obese groups had significantly higher pregestational BMI than NW, and OB GDM(+) showed significantly higher OGTT values than normoglycemic groups.

Gestational weight gain was lower in OB women, as suggested by the IOM (11.5–16 kg for NW; 5–9 kg for OB).

No significant differences were observed among groups for gestational age and fetal weight.

Placental efficiency (fetal weight/placental weight) was lower in the OB groups vs. NW, and OB women placentas were heavier and thicker compared to NW, though not significantly.

**Figure 1:**
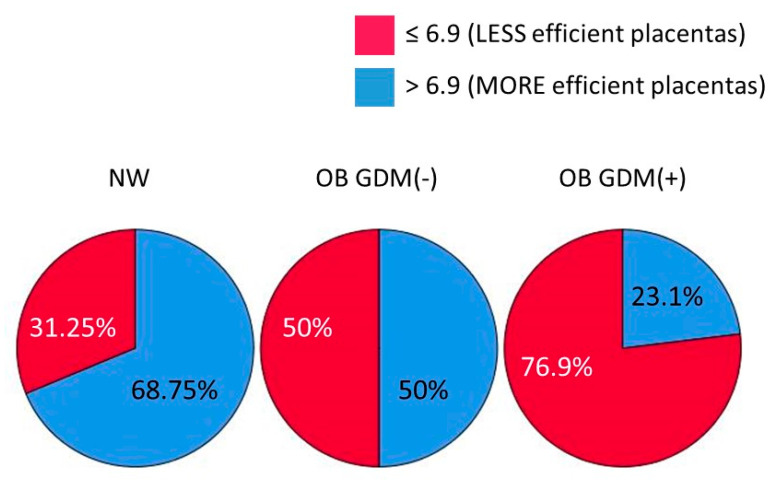
Distribution of placentas according to their efficiency for the three study groups (cut-off = 6.9, median value of placental efficiency). Data shown as a pie chart graph. Chi-square test: *p* = 0.049.

Interestingly, when using the median value of placental efficiency (6.9) as a cut-off to split placentas into subgroups of different efficiency, we found a significant difference in their distribution among the three study groups (chi-square test: χ2 (1, *n* = 47) = 6.001, *p* = 0.049, Φ = 0.36). Indeed, most of the placentas from NW women (68.75%), but only 23.1% of those from OB GDM(+) women, were in the “more efficient” subgroup. Differently, OB GDM(−) placentas were equally distributed in the two subgroups of efficiency (50%) (Figure 1).

A Two-way ANOVA was conducted to explore the impact of maternal characteristics (pregestational BMI and GDM) and fetal sex on these (i.e., fetal weight, placental weight and efficiency) parameters. The interaction effect between maternal BMI/GDM and fetal sex was not statistically significant. In the whole population and within each BMI group, no significant differences were found between male and female fetuses for these parameters.

Interestingly, when comparing the three BMI groups separately in female fetuses and male fetuses, placentas of OB GDM(+) women resulted in being significantly heavier and less efficient than NW in the female fetuses subgroup (Table 3).

### 3.2. Expression of Antioxidant Defense Genes in Placenta

To assess the profile of redox homeostasis-related genes in the placentas of NW, OB GDM(−) and OB GDM(+) women, we evaluated the gene expression level of the main modulators of the detox machinery.

The mRNA levels of CATalase (*CAT*), Glutathione SynthetaSe (*GSS*), Glutathione ReductaSe (*GSR*) and Glutathione PeroXidase 1 (*GPX1*) genes are reported in Table 4.

Placental expression was detected for all the analyzed antioxidant genes, showing lower levels of *CAT*, *GPX1* and *GSS* in the normoglycemic obese women vs. normal weight, though not significantly (Table 4).

Interestingly, although *CAT* did not reach statistical significance in the comparison between groups, its placental levels were significantly and positively correlated with *GSS* gene expression (r = +0.6, *p* < 0.001).

**Figure 2:**
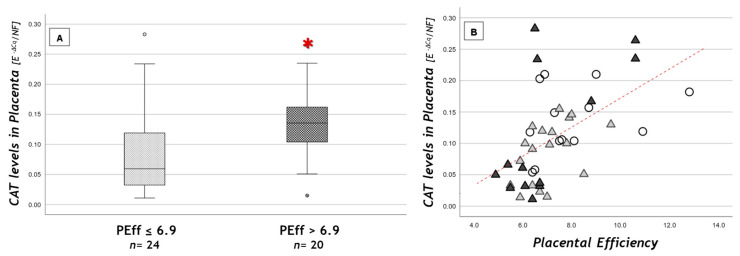
*CAT* placental levels (**A**) according to placental efficiency (PEff) cut-off and (**B**) in relation to PEff. (**A**) Data are shown as box plots, indicating the median and the 25th and 75th percentiles; ° values that extend more than 1.5 box-lengths from the edge of the box. * *p* < 0.05 (*t*-test); (**B**) statistical analysis by the Spearman rank order correlation (r = +0.6, *p* < 0.001) in NW (○), OB GDM(−) (▲), OB GDM(+) (▲).

When considering subgroups with different placental efficiency, *CAT* expression values were significantly higher in the “more efficient” placentas subgroup (efficiency >6.9) compared to the less efficient ones (≤6.9) (0.14 ± 0.06 vs. 0.09 ± 0.07; *p* = 0.019) (Figure 2A). Moreover, we found a strong positive correlation between placental efficiency and *CAT* levels (r = +0.6, *p* < 0.001) (Figure 2B). *CAT* expression values were also inversely correlated with placental weight and thickness (r = −0.61, *p* < 0.001 and r = −0.5, *p* = 0.001, respectively).

### 3.3. Autophagy-Related Gene Expression

To assess the autophagic processes, the expression of the genes encoding for the main drivers of the autophagic pathways was analyzed. The mRNA levels of Unc-51-Like Kinase 1 (*ULK1*), BECliN 1 (*BECN1*), Heat Shock Cognate 70 protein (*HSC70*), Lysosomal Associated Membrane Protein 2A (*LAMP-2A*) and Pleckstrin Homology domain and Leucine-rich repeat Protein Phosphatase 1 (*PHLPP1*) genes are reported in Table 5.

**Figure 3:**
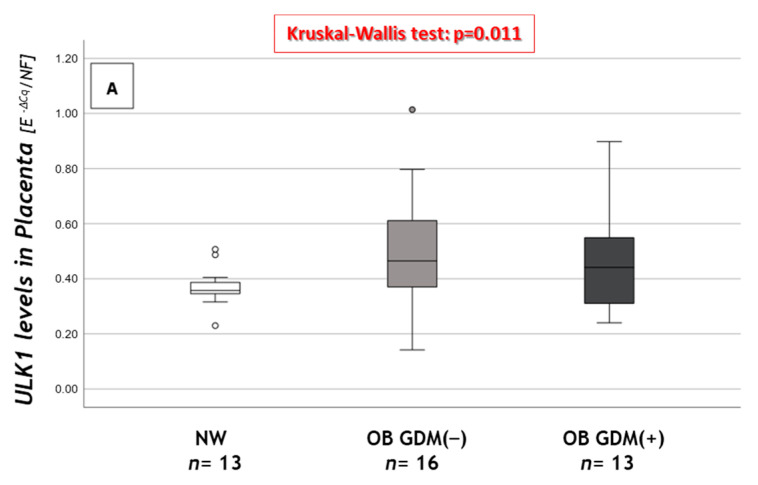

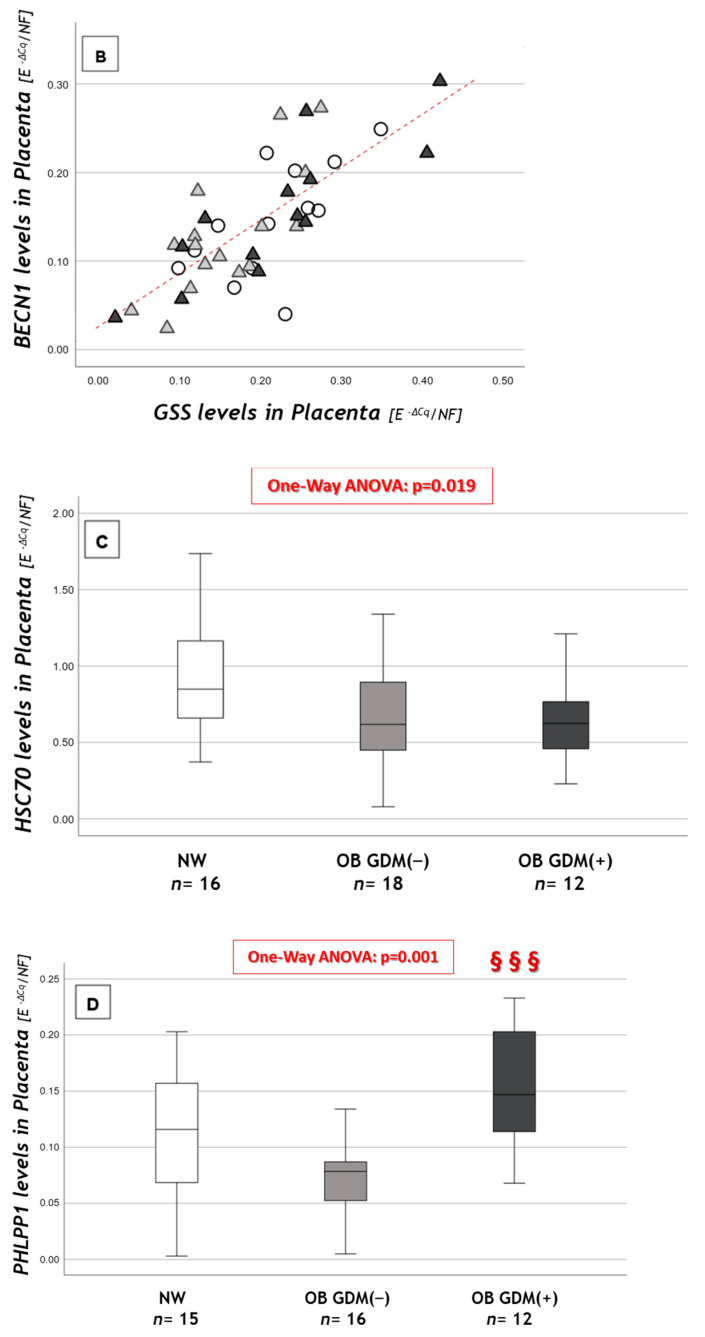
Expression of macroautophagy and CMA genes in placental tissue. (**A**) *ULK* gene expression levels, (**B**) correlation between *GSS* and *BECN1* placental levels, (**C**) *HSC70* gene expression levels and (**D**) *PHLPP1* gene expression levels among NW, OB GDM(−) and OB GDM(+). (**A**,**C**,**D**) Gene expressions were analyzed with Kruskal–Wallis test or One-way ANOVA (*ULK1*: *p* = 0.011; *HSC70*: *p* = 0.019; *PHLPP1*: *p* = 0.001). Data are shown as box plots, indicating the median and the 25th and 75th percentiles; ° values that extend more than 1.5 box-lengths from the edge of the box. **§§§** *p* ≤ 0.001 vs. OB GDM(−) (all Tukey HSD post hoc tests, except for *ULK* analyzed with Mann–Whitney U test and Bonferroni correction). (**B**) Statistical analysis by the Spearman rank order correlation (r = +0.76, *p* < 0.001) in NW (○), OB GDM(−) (▲), OB/GDM(+) (▲).

Significant results are reported in Figure 3.

As regards macroautophagy, *ULK1* showed significantly increased placental mRNA levels in the OB groups compared to NW (Figure 3A).

*BECN1* gene expression (Table 5) did not differ among the three groups. *BECN1* mRNA levels were significantly and strongly correlated with *GSS* levels (r = +0.76, *p* < 0.001) in the whole population (Figure 3B), and in each of its subgroups.

As regards chaperone-mediated autophagy (CMA), we measured the expression of genes that are involved at different levels in CMA activity or regulation.

*HSC70* expression was significantly different among groups. Indeed, both OB GDM(−) and OB GDM(+) presented lower levels compared to NW (Figure 3C).

*LAMP-2A* mRNA levels did not differ among groups (Table 5). In the NW subgroup, *LAMP-2A* placental levels were significantly and positively correlated with those of *GSS* (r = +0.8, *p* = 0.002).

In the obese subgroups, namely, OB GDM(−) and OB GDM(+), a significant positive correlation linked *LAMP-2A* and *HIF-1α* placental expressions (r = +0.67, *p* < 0.001).

*PHLPP1* expression was significantly different among groups. Post hoc analysis showed that its levels were significantly increased in OB GDM(+) compared to OB GDM(−) placentas (Figure 3D).

When considering the NW subgroup, *PHLPP1* placental levels were significantly correlated with *HSC70* expression (r = +0.53, *p* = 0.034), while this correlation was not significant in both OB GDM(−) and OB GDM(+) groups.

Finally, the expression of genes encoding proteins involved in the cell response to stressors and related to CMA activity was evaluated (Table 6): Nuclear factor erythroid 2-Related Factor 2 (*NRF2*), Hypoxia-Inducible Factor 1 (*HIF-1α*), Vascular Endothelial Growth Factor (*VEGF*).

*NRF2*, *HIF-1α* and *VEGF* mRNA levels did not differ among the three groups (Table 6).

**Figure 4:**
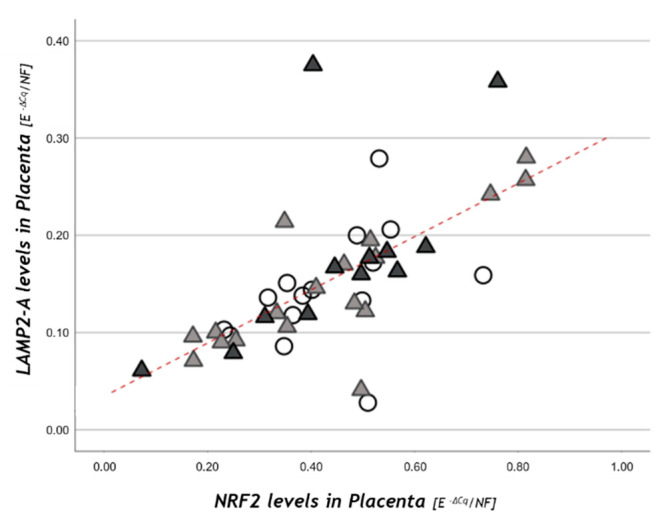
*NRF2* placental levels in relation to *LAMP-2A*. Statistical analysis by the Spearman rank order correlation (r = +0.73, *p* < 0.001) in NW (○), OB GDM(−) (▲), OB/GDM(+) (▲).

*NRF2* placental levels resulted in being significantly correlated with *LAMP-2A* in the study population (r = +0.73, *p* < 0.001; Figure 4) and within each study group.

### 3.4. Sexually Dimorphic Placental Expressions

A chi-square for independence test (with continuity correction) indicated no significant association between fetal sex and body mass index (χ2 (1, *n* = 47) = 0.31, *p* = 0.86, Φ = 0.08). Indeed, fetal sex frequencies did not differ among the three study groups (Males- M: 56.25% of NW, 50% of OB GDM(−) and 46.2% of OB GDM(+); Females- F: 43.75% of NW, 50% of OB GDM(−) and 53.8% of OB GDM(+)).

To understand if there was a possible joint effect of fetal sex and maternal metabolic characteristics (BMI and maternal glycemia) on levels of the analyzed genes, we performed additional analyses.

A Two-way ANOVA explored the interaction effect of maternal BMI/hyperglycemia and fetal sex on our molecular variables, but no statistical significance was found.

A comparison among the three BMI groups was then performed in female or male placentas, using non-parametric statistics due to the sample sizes of the fetal sex subgroups.

In female, but not in male, placentas, a lower expression was recorded for *CAT*, *GSS* and *GPX1* in OB GDM(−) (*CAT*: 0.070 ± 0.048; *GSS*: 0.133 ± 0.056; *GPX1*: 0.141 ± 0.062) vs. NW (*CAT*: 0.134 ± 0.040; *GSS*: 0.213 ± 0.368; *GPX1*: 0.212 ± 0.660), though not significantly.

Macroautophagy genes did not show any difference when dividing by both BMI/GDM and fetal sex.

Among chaperone-mediated autophagy-related genes, similarly to antioxidant enzymes, a lower *HSC70* expression was found in OB GDM(−) (0.585 ± 0.234) compared to NW (0.886 ± 0.237) when analyzing placentas from female fetuses.

**Figure 5:**
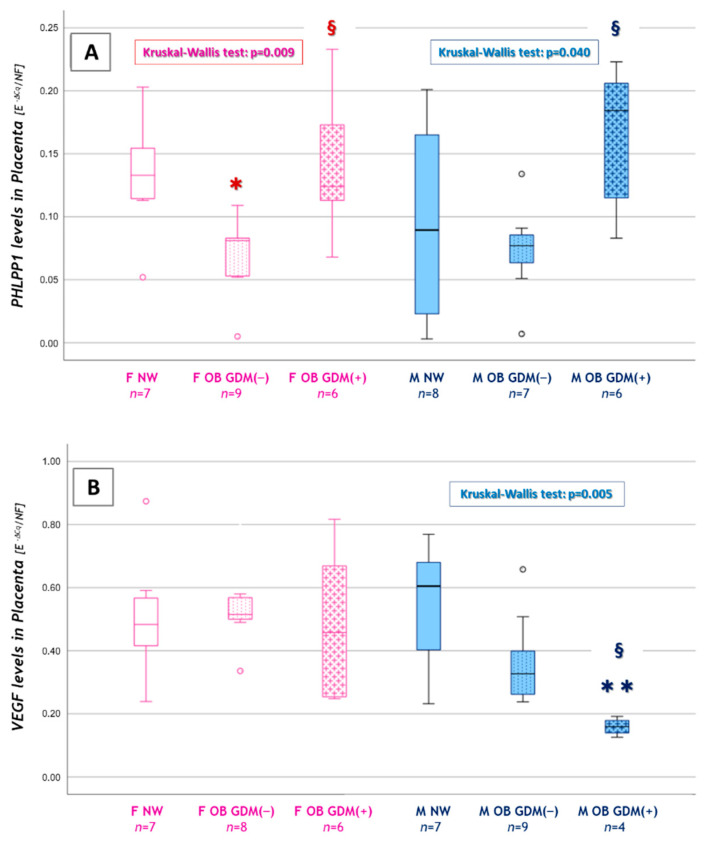
Sexually dimorphic expression of CMA or autophagy-related genes. (**A**) *PHLPP1* and (**B**) *VEGF* levels among female or male placentas in NW, OB GDM(−) and OB GDM(+). Data were analyzed with Kruskal–Wallis test (*PHLPP1*: *p* = 0.009 for FEMALE placentas, *p* = 0.040 for MALE placentas. *VEGF*: *p* = 0.005 for MALE placentas). Data are shown as box plots, indicating the median and the 25th and 75th percentiles; ° values that extend more than 1.5 box-lengths from the edge of the box. FEMALE Placentas: * *p* < 0.05 vs. NW or § *p* < 0.05 vs. OB GDM(−). MALE Placentas: ** *p* < 0.01 vs. NW or § *p* < 0.05 vs. OB GDM(−); post-hoc by Mann–Whitney U test with Bonferroni correction.

Significantly lower values were observed for *PHLPP1* in females’ placentas (OB GDM(−): 0.069 ± 0.317 vs. NW: 0.132 ± 0.048; Figure 5A). Moreover, in placentas from female fetuses, *PHLPP1* levels resulted in being significantly increased in OB GDM(+) (0.139 ± 0.057) compared to normoglycemic obese mothers.

Interestingly, *PHLPP1* levels were significantly increased also in males’ placentas, in OB GDM(+) (0.166 ± 0.056) compared to OB GDM(−) (0.073 ± 0.039) (Figure 5A). An increased, though not significant, *LAMP-2A* expression was also observed in males’ placentas, in OB GDM(+) (0.226 ± 0.111) compared to NW (0.147± 0.045).

Finally, a significant reduction in *VEGF* expression was observed in males’ placentas in the OB GDM(+) group (0.159 ± 0.027) when compared to both OB GDM(−) (0.365 ± 0.140) and NW (0.539 ± 0.212). *VEGF* gene expression was also lower, though not significantly, in OB GDM(−) male placentas vs. NW (Figure 5B).

## 4. Discussion

This work focused on the description of placental function and molecular features related to cell response to oxidative stress and autophagy, in the context of metabolic deregulation characterizing maternal obesity and gestational diabetes. Indeed, placentas obtained from normal weight and obese women with or without gestational diabetes were analyzed for the expression of different gene subsets.

We found a decrease, though not significant, of placental antioxidant defense genes (*CAT*, *GSS*, *GPX1*) in OB GDM(−), but not in OB GDM(+), compared to NW. Interestingly, *CAT* was strongly correlated with placental efficiency and had significantly lower levels in less efficient placentas. Among genes playing an important role in macroautophagy, placental *ULK1* was increased in OB groups compared to NW, while *BECN1* presented a strong and positive correlation with *GSS* gene expression. The chaperone-mediated autophagy regulator *PHLLP1* was reduced in OB GDM(−) placental tissues compared to NW and significantly increased in OB GDM(+) compared to OB GDM(−). *PHLLP1* also correlated with *HSC70* in NW placental tissues, but not in OB populations. Other CMA-related genes (*NRF2*, *HIF-1a*, *VEGF*) did not show significant differences in obesity and GDM.

When analyzing placental gene expression in relation to fetal sex, a sexual dimorphism was found for both antioxidant and CMA-related genes.

Importantly, in this study, placentas derived only from elective cesarean section, i.e., in the absence of labor, were included. This allowed avoiding any bias related to labor-induced oxidative stress, inflammation and changes in cell metabolism.

Furthermore, the clinical characteristics of the study population were deeply characterized: patients were carefully selected, and both NW and OB groups did not present any associated pathology, except for GDM in OB GDM(+). We excluded any maternal or fetal infection or autoimmune disease, maternal drug–alcohol abuse, fetal malformations, chromosomal disorders, preeclampsia and intrauterine growth restriction, all of which can affect the oxidative and the inflammatory status [28,29,30,31]. Moreover, only Caucasian women were selected, as different metabolic and oxidative characteristics have been identified and have been suggested to possibly contribute to the genetic component of complex disorders [32,33]. Our cases were also carefully matched to NW controls with similar characteristics except for BMI and GDM presence. Finally, all women included in this study were counseled with nutritional and lifestyle advice and recommendations on weight gain during pregnancy, and obese patients had regular specific checkups in a dedicated antenatal clinic with specific dietary indications. Therefore, we may assume that the reported findings can be related to metabolic dysfunctions related to increased BMI or hyperglycemic status [34].

Placental efficiency, calculated as fetal/placental weight, was lower in both obese groups compared to NW. When using the median value of placental efficiency as a cut-off, the percentage of the most efficient placentas (above the cut-off) decreased progressively, moving from the normal weight population to the obese and obese with GDM women. Since a decrease in placental efficiency has been described in relation to the oxidative stress induced by obesity [25,35], the expression of genes coding for proteins involved in cell redox homeostasis was analyzed in the different groups.

Expression of antioxidant defense genes (*CAT*, *GSS*, *GSR*, *GPX1*) tended to decrease in OB GDM(−) compared to NW. This trend supports an increase in oxidative stress and the evidence of altered placental metabolites involved in antioxidant defenses that has been previously described in the obese population [6,7,36].

Interestingly, expression levels of these genes in GDM women appeared to be more similar to those of NW. This might be due to different responses in the obese and diabetic impaired environment [37], leading to alternative mechanisms that result in an unchanged expression of the analyzed antioxidant genes. Indeed, previous studies showed that while placentas from obese women without comorbidities presented impaired mitochondrial biogenesis and respiratory chain enzyme activities [31], these were not altered in OB GDM(+) placentas, although mitochondrial morphological abnormalities were shown, accounting for mitochondrial dysfunctionality [7,38]. Moreover, we previously reported a differential alteration in antioxidant metabolites’ content in OB placentas, depending on the presence or absence of GDM, showing different profiles in these two groups [36].

The *CAT* gene aroused particular attention because of its strong correlation with placental efficiency. In fact, in most efficient placentas, a significant increase in *CAT* expression was found, suggesting the importance of the redox potential in the maintenance of organ functionality [39].

Another cellular mechanism involved in the maintenance of cell homeostasis is autophagy. Importantly, reactive oxygen species are master inducers of autophagy. Thus, the activation of this process is a key part of the cellular response to oxidative stress because it allows scavenging of defective components before further damage [9,40].

Autophagy activity has been documented in placentas, but only a few studies have investigated it in obese and diabetic placentas [20,21,41]. Autophagy includes different degradation systems, mainly divided into unspecific and selective strategies. The former group is mainly represented by macroautophagy, which acts by including cytoplasmic material and organelles within an autophagosome whose fusion with a lysosome induces the degradation of the content [42]. On the other hand, the selective strategies such as chaperone-mediated autophagy degrade only selected targets containing a specific consensus sequence called KFERQ. This motif is usually recognized and bound by the chaperone Heat Shock Cognate 70 (HSC70); the complex is then delivered to the lysosome where the protein, upon its interaction with the LAMP-2A multimer, forming a channel on the lysosomal membrane, is unfolded and enters the lysosome where it will be digested by different proteases [43].

The Unc-51-Like Kinase 1 (ULK1) complex plays a central role in the macroautophagy initiation stage and is also involved in promoting autophagosome–lysosome fusion [44]. Interestingly, our study showed an increase in *ULK1* expression in the placentas of OB groups, possibly due to the metabolic stress caused by obesity and activating macroautophagy. This phenomenon correlates with the already mentioned expression reduction in genes involved in the detoxification from oxygen reactive species, inducing macroautophagy.

Another main player of macroautophagy is BECliN 1 (BECN1), being the principal driver of this degradative pathway [45]. Even if no statistically significant changes were observed in the three populations, a strong and positive correlation with *GSS* gene expression was noted in placentas and was sustained in each subgroup, as a clue of the relation between autophagy and the cell response to oxidative stress [46].

On the other hand, the chaperone-mediated autophagy machinery showed some changes in the expression of its players. In detail, *HSC70* expression decreased in obese mothers, and also in the presence of diabetes, compared to normal weight, indicating a potential impairment in CMA activity. *LAMP-2A* expression did not change among groups. However, *LAMP-2A* positively correlated with *GSS* in NW, accounting for the expected oxidative stress-induced CMA activity in the control group. This correlation was not significant in OB GDM(−) and OB GDM(+). Nevertheless, a positive correlation between *LAMP-2A* and *HIF-1α* (a well-known CMA target) emerged only in OB placentas, suggesting a different activity of CMA in obesity. However, this hypothesis needs further investigations.

CMA is finely regulated by several proteins, including Pleckstrin homology domain and leucine-rich repeat protein phosphatase 1 (PHLPP1) [47]. This phosphatase is able to induce CMA activity by favoring the stabilization of the LAMP-2A multimers in the lysosomal membrane. Moreover, this protein has been described to be also involved in insulin resistance mechanisms [48,49]. Indeed, it supports insulin resistance by reducing insulin-dependent signal transduction, leading to a decrease in glucose transport and hyperglycemia [50].

In our study, *PHLLP1* expression was reduced in OB GDM(−) placental tissues compared to NW, while it resulted in being significantly increased in OB GDM(+) compared to OB GDM(−), supporting a correlative hypothesis between PHLPP1 activity and GDM, as already hypothesized in type 2 diabetes by Andreozzi and colleagues [49]. Moreover, a positive and strong correlation between *PHLPP1* and *HSC70* was observed in NW placental tissues, but not in OB populations, again supporting the hypothesis of an impaired CMA function in obesity. This interesting result could suggest a role for PHLPP1 in GDM placentas. However, whether it has a causative or a secondary role in relation to insulin resistance still needs to be investigated [48]. In fact, it has been described that PHLPP1 overexpression induces insulin resistance. However, an increase in insulin levels also resulted in the overexpression of PHLPP1 itself, being probably involved in a regulating feedback loop that is lost in diabetes, for reasons that are not yet understood.

The expression of other genes whose transcription is triggered by stressors or modulated by CMA activity (*NRF2*, *HIF-1α*, *VEGF*) was also studied and did not show significant differences in obesity and GDM. Among these, NRF2 plays an important role in driving the transcription of different genes involved in cell detoxification and is also related to CMA activation because its activity is directly related to LAMP-2A expression [51]. Data reported herein confirm the strong positive correlation between *NRF2* and *LAMP-2A* in each group.

We also explored the possibility of significant differences in relation to fetal sex. Indeed, different responses to an adverse intrauterine environment have been previously extensively documented depending on fetal sex [52,53,54,55].

We analyzed fetal weight, placental weight and placental efficiency in males and females across BMI groups. When considering female newborns, the diabetic obese placentas resulted in being heavier, with a significantly decreased efficiency when compared to normal weight. These results are supported by our previous reports in diabetic obese placentas [7,55], thus suggesting interesting evidence of sexual dimorphism in placental biometrical and functional features [21].

**Figure 6:**
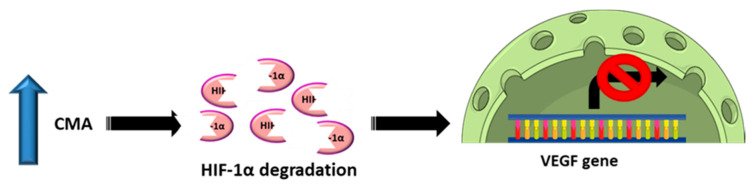
Schematic representation of our hypothesis connecting the regulation of VEGF expression and chaperone-mediated autophagy (CMA) activity.

An interesting result was also obtained for the expression of *PHLPP1*, which dropped significantly in females’ placentas in the OB GDM(−) group, while it increased in both male and female placentas in OB GDM(+). However, *PHLPP1* modulation seemed to have different consequences in the two sexes, with a significant decrease in *VEGF* expression only in males, which might be due to CMA activation. In fact, HIF-1α is both a CMA target and one of the main transcription factors driving VEGF expression (Figure 6). Supporting the hypothesis of a CMA activation, an increase in the expression of its main player, *LAMP-2A*, has been detected in the same samples even if it did not reach statistical significance. This mechanism was not observed in females’ tissues, providing clues about a dimorphic regulation and possibly different GDM outcomes depending on fetal sex.

## 5. Conclusions

In this study, we presented preliminary data on the expression of antioxidant and autophagy-related genes in placentas from normal weight and obese mothers, with or without gestational diabetes mellitus. Despite the small population size, which limited the statistical significance of some modulations, our analysis showed intriguing results, reporting differential alterations depending on the presence/absence of GDM and on fetal sex. These results confirm how metabolism, nutrient availability and cellular mechanisms such as OxS responses and autophagy are strictly connected. Further studies will be necessary to unravel processes below this observation. In detail, the role of PHLPP1 and chaperone-mediated autophagy activation in the etiopathology and also possibly in adverse outcomes of GDM might be suggested, deserving further investigations.

In the context of the current research, to our knowledge, this is the first report describing an increase in *PHLPP1* expression in placentas in relation to GDM. New studies are needed to understand the mechanisms underlying this deregulation. Their comprehension could be useful for preventing the development of GDM. The elucidation of PHLPP1 involvement will pave the way for further analyses aimed at explaining its role in placental alterations in the context of metabolic disorders and its potential targetability for the treatment of negative consequences of diabetes.

## Figures and Tables

**Table 1:** Primer details.

Gene	Forward Sequence (5′–3′)	Reverse Sequence (3′–5′)	Fw Position *	Rev Position *	Amplicon Length
*YWHAZ*	ACTTTTGGTACATTGTGGCTTCAA	CCGCCAGGACAAACCAGTAT	1110–1133	1203–1184	51 bp
*β*–*ACTIN*	ATCAAGATCATTGCTCCTCCTGA	CTGCTTGCTGATCCACATCTG	1063–1085	1164–1144	59 bp
*CAT*	TAAGACTGACCAGGGCA	CAAACCTTGGTGAGATCGAA	788–804	988–969	165 bp
*GSS*	ATGCTGTGCAGATGGACTTCAACC	TGGATGTCAAACAGACGAGCGGTA	285–308	408–385	77 bp
*GSR*	AACATCCCAACTGTGGTCTTCAGC	TTGGTAACTGCGTGATACATCGGG	1239–1262	1378–1355	93 bp
*GPX1*	CGCAACGATGTTGCCTGGAACTTT	AGGCTCGATGTCAATGGTCTGGAA	563–586	664–641	55 bp
*ULK1*	TCATCTTCAGCCACGCTG	CACGGTGCTGGAACATCT	2724–2741	2789–2772	31 bp
*BECN1*	GGCTGAGAGACTGGATCAGG	CTGCGTCTGGGCATAACG	806–825	932–915	90 bp
*HSC70*	ATTGATCTTGGCACCACCTA	ACATAGCTTGAAGTGGTTCG	103–122	203–184	62 bp
*LAMP–2A*	TGCTGGCTACCATGGGGC TG	GCAGCTGCCTGTGGAGTGAGT	825–844	922–902	58 bp
*PHLPP1*	CCTACCTTCTCCAGTGCACT	CCAGCAGTTCCAAGTTTCCT	3796–3815	3916–3897	82 bp
*NRF2*	CAGCGACGGAAAGAGTATGA	AAGAAACCTGGGAGTAG	271–291	471–454	163 bp
*HIF–1* *α*	TGATTGCATCTCCATCTCCTACC	GACTCAAAGCGACAGATAACACG	2205–2227	2381–2359	132 bp
*VEGF*	CGAGGGCCTGGAGTGTGT	CGCATAATCTGCATGGTGATG	335–352	391–371	19 bp

Table 1 shows the primer sequences (forward and reverse) used to amplify all the genes, their positions and the length of amplicons. * https://blast.ncbi.nlm.nih.gov/Blast.cgi. (Accessed on 30 March 2021).

**Table 2:** Maternal, fetal and placental data.

	One-Way ANOVA or Kruskal–Wallis Test	NW *n* = 16	OB GDM(−) *n* = 18	OB GDM(+) *n* = 13
**Maternal Data**				
Age (years) ^A^	*p* = 0.002	35.7 ± 3.5	30.2 ± 6.0 **	35.8 ± 4.3 §§
Pregestational BMI (kg/m^2^) ^B^	*p* = 0.000	21.18 ± 2.11	34.80 ± 4.06 ***	35.05 ± 3.41 ***
OGTT I Value (mg/dL) ^A^	ns	84.00 ± 5.12	79.92 ± 6.52	89.73 ± 15.08
OGTT II Value (mg/dL) ^A^	*p* = 0.000	117.73 ± 19.14	113.10 ± 22.57	164.67 ± 35.99 *** §§§
OGTT III Value (mg/dL) ^B^	*p* = 0.001	105.36 ± 36.28	92.20 ± 14.19	162.89 ± 32.28 ** §§§
Gestational Weight Gain (kg) ^A^	ns	10.93 ± 2.86	8.44 ± 6.06	6.62 ± 5.91
Hemoglobin (mg/dL), at 34–36 weeks ^B^	ns	12.13 ± 1.16	11.35 ± 0.76	11.37 ± 0.97
**Fetal (F) and Placental (P) Data at Delivery**				
Gestational Age (weeks) ^B^	ns	39.26 ± 0.50	39.16 ± 0.30	39.09 ± 0.16
Fetal Weight (g) ^A^	ns	3337.94 ± 247.71	3339.44 ± 417.17	3330.00 ± 336.54
Placental Weight (g) ^A^	ns	434.25 ± 80.43	482.22 ± 81.50	497.46 ± 102.55
Placental Surface (cm^2^) ^A^	ns	245.59 ± 45.25	232.34 ± 85.03	245.44 ± 46.41
Placental Thickness (cm) ^A^	ns	1.81 ± 0.42	2.21 ± 0.58	2.04 ± 0.55
Placental Efficiency (F/P weight) ^B^	ns	7.97 ± 1.75	7.04 ± 1.04	6.98 ± 1.85

Data were analyzed according to their distribution with ^A^. One-way ANOVA or ^B^. Kruskal–Wallis test. Data are shown as mean ± standard deviation; ** *p* < 0.01, *** *p* ≤ 0.001 vs. Normal Weight (NW); §§ *p* < 0.01, §§§ *p* ≤ 0.001 vs. Obese without GDM (OB GDM(−)) refer to post hoc analyses, performed depending on data distribution by: ^A^. Tukey’s HSD test or ^B^. Mann–Whitney U test with Bonferroni correction. BMI: body mass index; OGTT: Oral Glucose Tolerance Test (OGTT I: fasting glycemic value; OGTT II: glycemic value after 60 min from 75 g glucose consumption; OGTT III: glycemic value after 120 min from 75 g glucose consumption).

**Table 3:** Fetal and placental characteristics separately described across sexes.

	MALE FETUSES (*n* = 24)				FEMALE FETUSES (*n* = 23)			
	One-Way ANOVA or Kruskal–Wallis Test	NW *n* = 9	OB GDM(−) *n* = 9	OB GDM(+) *n* = 6	One-Way ANOVA or Kruskal–Wallis Test	NW *n* = 7	OB GDM(−) *n* = 9	OB GDM(+) *n* = 7
Fetal (F) Weight (g) ^A^	ns	3371.67 ± 287.26	3368.89 ± 450.68	3374.17 ± 369.49	ns	3294.57 ± 198.68	3310.00 ± 405.90	3292.14 ± 283.58
Placental (P) Weight (g) ^A^	ns	451.44 ± 79.26	495.56 ± 95.93	441.17 ± 105.75	*p* = 0.011	412.14 ± 82.35	468.89 ± 67.17	545.71 ± 76.35 **
Placental Efficiency (F/P weight) ^B^	ns	7.76 ± 2.02	6.92 ± 0.89	8.05 ± 2.27	*p* = 0.012	8.24 ± 1.46	7.16 ± 1.22	6.07 ± 0.68 **

Data were analyzed according to their distribution with ^A^. One-way ANOVA or ^B^. Kruskal–Wallis test. Data are shown as mean ± standard deviation; ** *p* < 0.01 vs. NW refers to post hoc analyses, performed depending on data distribution by: ^A^. Tukey’s HSD test or ^B^. Mann–Whitney U test with Bonferroni correction.

**Table 4:** Gene expression of antioxidant defense enzymes in placental tissue.

Antioxidant Defenses	NW *n =* 16	OB GDM(−) *n =* 18	OB GDM(+) *n =* 13
*CAT* ^A^	0.146 ± 0.054	0.087 ± 0.048	0.115 ± 0.104
*GSS* ^A^	0.215 ± 0.071	0.159 ± 0.068	0.218 ± 0.114
*GSR* ^B^	0.265 ± 0.131	0.213 ± 0.116	0.294 ± 0.180
*GPX1* ^A^	0.207 ± 0.067	0.152 ± 0.062	0.187 ± 0.061

Data are shown as mean ± standard deviation. Data were analyzed depending on their distribution with: ^A^. One-way ANOVA or ^B^. Kruskal–Wallis test.

**Table 5:** Expression levels of macroautophagy and chaperone-mediated autophagy (CMA) genes in placental tissue.

Macroautophagy and CMA	One-Way Anova	NW *n =* 16	OB GDM(−) *n =* 18	OB GDM(+) *n =* 13
*BECN1* ^A^	ns	0.145 ± 0.063	0.142 ± 0.088	0.155 ± 0.078
*LAMP-2A* ^A^	ns	0.143 ± 0.058	0.147 ± 0.068	0.179 ± 0.097

Data were analyzed according to their distribution with ^A^. One-way ANOVA. Data are shown as mean ± standard deviation.

**Table 6:** Expression levels of autophagy-related genes in placental tissue.

Autophagy-Related Genes	NW *n =* 16	OB GDM(−) *n =* 18	OB GDM(+) *n =* 13
*NRF2* ^A^	0.433 ± 0.128	0.437 ± 0.203	0.449 ± 0.181
*HIF-1α* ^B^	0.235 ± 0.080	0.275 ± 0.168	0.218 ± 0.087
*VEGF* ^A^	0.524± 0.198	0.447 ± 0.158	0.354 ± 0.243

Data are shown as mean ± standard deviation. Data were analyzed depending on their distribution with: ^A^. One-way ANOVA or ^B^. Kruskal–Wallis test.

## Data Availability

All data that support the findings of this study are available from the corresponding author (L.O. and C.M.) on reasonable request.
